# iBench: A ground truth approach for advanced validation of mass spectrometry identification method

**DOI:** 10.1002/pmic.202200271

**Published:** 2022-10-17

**Authors:** John A. Cormican, Wai Tuck Soh, Michele Mishto, Juliane Liepe

**Affiliations:** ^1^ Max‐Planck‐Institute for Multidisciplinary Sciences (MPI‐NAT) Göttingen Germany; ^2^ Centre for Inflammation Biology and Cancer Immunology (CIBCI) & Peter Gorer Department of Immunobiology King's College London London UK; ^3^ The Francis Crick Institute London UK

**Keywords:** benchmarking, HLA, immunopeptidome, method performance, proteomics

## Abstract

The discovery of many noncanonical peptides detectable with sensitive mass spectrometry inside, outside, and on cells shepherded the development of novel methods for their identification, often not supported by a systematic benchmarking with other methods. We here propose iBench, a bioinformatic tool that can construct ground truth proteomics datasets and cognate databases, thereby generating a training court wherein methods, search engines, and proteomics strategies can be tested, and their performances estimated by the same tool. iBench can be coupled to the main database search engines, allows the selection of customized features of mass spectrometry spectra and peptides, provides standard benchmarking outputs, and is open source. The proof‐of‐concept application to tryptic proteome digestions, immunopeptidomes, and synthetic peptide libraries dissected the impact that noncanonical peptides could have on the identification of canonical peptides by Mascot search with rescoring via Percolator (Mascot+Percolator).

**Abbreviations**
FDR: false discovery rate
HLA: human leukocyte antigen
iBench: in silico BENChmark Helper
MS: mass spectrometry
ORF: open reading frame
PTM: post‐translational modifications
PSM: peptide spectrum match
PR: precision recall
ROC: receiver operator characteristic
UTR: untranslated region

## 1 INTRODUCTION

In the last decade, the striking progress in mass spectrometry (MS) has triggered the development of methods that looked behind the curtain of conventional peptidomics and proteomics. As a consequence, a plethora of ‘noncanonical’ peptides and polypeptides have been identified both inside the cell, inside cell compartments, in the extracellular space, as well as in the cleft of the Human Leukocyte Antigen class (HLA‐) I and II, also known as HLA‐I and HLA‐II immunopeptidomes. The ‘noncanonical peptides’ can deviate from canonical peptides in terms of features, origin, and mechanism of generation [1].

Canonical peptides may be generated via peptide hydrolysis by proteases either in physiological conditions such as proteasome proteolysis *in cellula* or in technical reactions such as tryptic digestions of intracellular protein content, also known as intracellular proteome. These peptides derive from known proteins included in proteome databases such as the human UniProt reference proteome. Noncanonical peptides include those that underwent post‐translational modifications (PTMs) and/or are not derived from proteins listed in canonical proteome databases. PTMs of proteins and peptides can be achieved via chemical modifications either *in cellula* (biological PTMs) or during the preparation of the samples and MS measurement (technical PTMs). Other kinds of PTMs modify the amino acid sequence of peptides and proteins. Although often these reactions are catalyzed by enzymes (e.g., transpeptidation that generates *cis*‐ and *trans*‐spliced peptides), also enzyme‐independent reactions‐, for example, protein splicing of inteins ‐ can occur [2]. A large portion of noncanonical peptides may derive from putative non‐coding regions‐, for example, 5′‐UTR, 3′‐UTR, introns ‐ or alternative transcription and translation processes, such as alternative open reading frame (ORF) usage, alternative RNA splicing, and ribosomal frameshift. These peptides are defined as ‘cryptic’ [3, 4].

From the proteomics point of view, the identification of noncanonical peptides could not be achieved with standard methods. Novel tools have been developed to this end (e.g., [3, 5‐9]), and new challenges to the statistics underlying proteomics confidence estimation have emerged. In particular, the estimation of false discovery rate (FDR), which is the cornerstone of many proteomics search engines, became problematic since the theoretical database of noncanonical peptides has been estimated to be dramatically larger than that of the canonical peptides.

To address this issue, noncanonical peptide candidates identified in various sample types such as tryptic proteome digestions and HLA‐I immunopeptidomes have been investigated at MS2 spectral level by predicting MS2 spectra and comparing them with the measured ones (e.g., [7, 10‐13]), and at peptide level by comparing canonical and noncanonical peptides’ features (e.g., [3, 14].

These attempts assumed a similarity between the features of canonical and noncanonical peptides. In contrast, differences in the mechanism of production, features of the original proteins (among others) between canonical and noncanonical peptides have been hypothesized [3‐5, 15‐17], and therefore this strategy could be biased. To tackle this issue, we could synthesize all noncanonical peptides identified in a proteomic sample, measure them via MS and compare the MS2 spectra of the peptides and synthetic peptides, which has only been undertaken rarely [18].

Although the comparison of MS2 spectra and peptide features could help identifying sequence misassignment, and thus improve the precision of a method, it could not estimate or improve the recall of a method, that is, the proportion of peptides of a given kind that were present in a sample but were not identified by a given method. By neglecting this aspect, the low recall of a method could confound the identification of peptides of a given kind, thereby leading to lower estimates of the frequency of that specific peptide kind [19]. The generation of a ground truth dataset containing peptide spectrum matches (PSMs) with characteristics similar to the target datasets, and the benchmarking of a given method on that dataset could address both issues. Indeed, this approach could let us estimate the precision‐, that is, number of correctly identified peptides over number of identified peptides –and recall‐, that is, number of correctly identified peptides over number of all correct peptides in the sample – of a given method. The computation of precision and recall (PR) and receiver operating characteristic (ROC) curves is a standard strategy for performance evaluation of in silico predictors.

**FIGURE 1:**
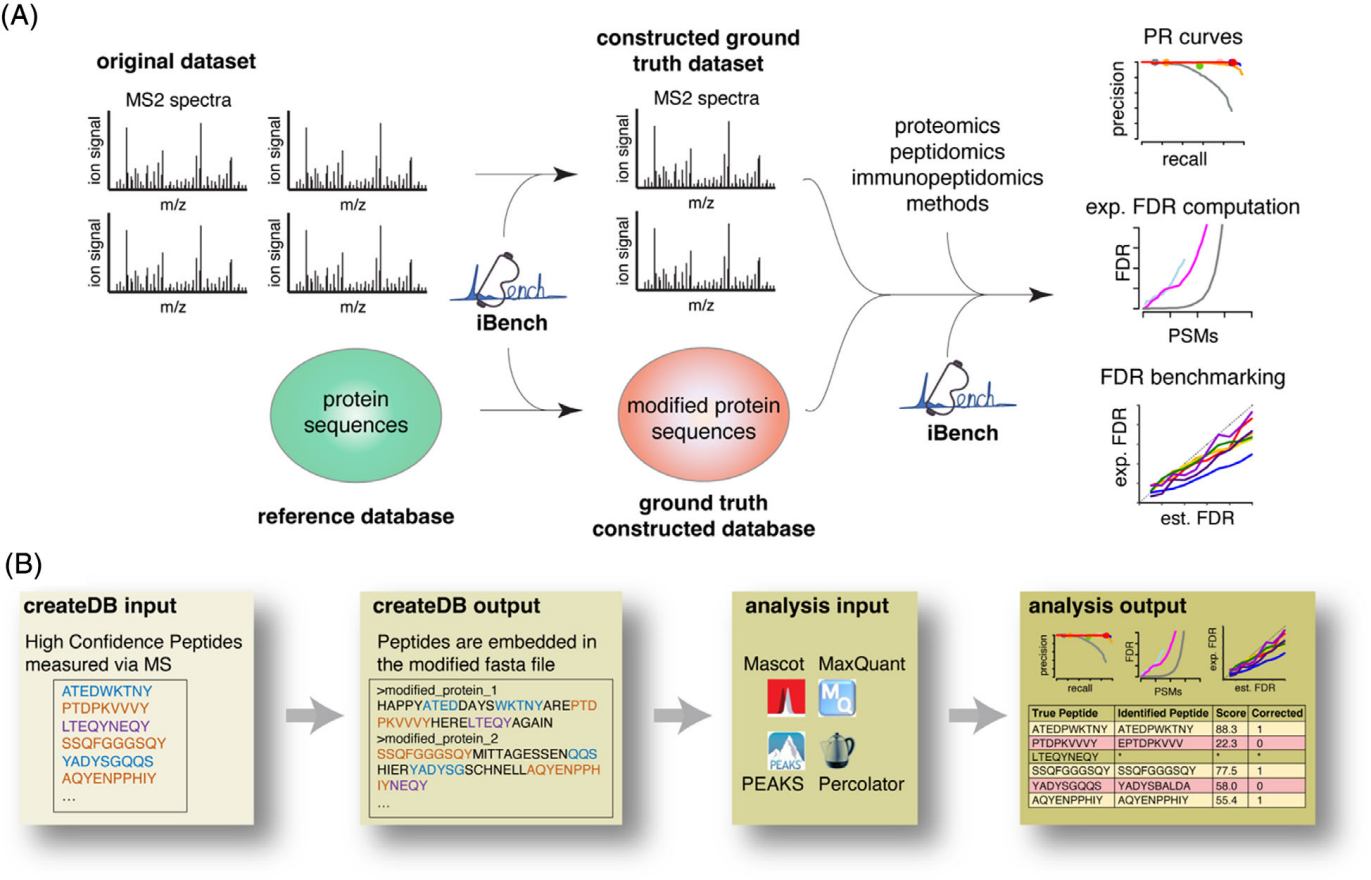
iBench and benchmarking workflow. (A) Overview of the processing and data flow through the iBench “createDB” and “analysis” workflows. (B) Flow chart representing the inputs and outputs of the iBench “createDB” and “analysis” workflows.

Recently, we applied this strategy to compare the impact of database search engines and target database features on the identification of post‐translationally spliced peptides in HLA‐I immunopeptidomes. We manually created ground truth datasets and cognate databases, and benchmarked PR performance of Mascot, Mascot+Percolator and PEAKS as final search engines [20]. The identification of post‐translationally spliced peptides, which can be generated by proteasomes as well as other proteases and trigger an immune response [21], is an emblematic field wherein the automated creation of ground truth datasets and databases could favor an educated evaluation of the performance of novel methods, and provide a catharsis to the debate on the natural existence of this process [19, 22]. To this end, we developed the software package in silico *BENChmark Helper* (iBench). It constructs ground truth proteomics datasets and reference databases, which can be used to test methods, search engines, and proteomics strategies. iBench also provides a pool of standard performances outputs such as PR and ROC curves. iBench is open source, can be combined with various search engines and is not computationally demanding or convoluted (Figure 1).

As proof‐of‐concept, we applied iBench to tryptic proteome digestions, HLA‐I immunopeptidomes and synthetic peptide libraries (see Table 1) to estimate how noncanonical peptides present in the datasets could impinge upon the successful identification of canonical peptides by Mascot+Percolator, and to compare estimated and measured FDRs. In this representative application, noncanonical peptides were unidentifiable by standard Mascot+Percolator, and therefore represented a ‘stress‐test’ for the investigated search engine, and the impact on its performance could be quantified by applying iBench.

**TABLE 1:** Benchmarking strategies using Bench to generate ground truth datasets

Source of ground truth datasets	Interpretation	Advantages
High confidence PSMs from a standard search	Internal Consistency of Search Engine	Easy validation.
		Similarity to target peptides.
High confidence PSMs from multiple search engines	Internal Consistency and Removal of Search Engine Specific Bias.	Easy validation.
		Similarity to target peptides.
Synthetic peptide library measurements	True Precision‐Recall	High confidence in identifications.
		Suitable for benchmarking of very precise tools.

## 2 MATERIALS AND METHODS

### 2.1 Cell lines

K562‐A*02:01 cell clone expresses single HLA‐I alleles. It derives from the leukemia K562 cell line (ATCC CCL‐243), which does not express endogenous HLA‐I and ‐II molecules. K562‐A*02:01 cell clone generation is described elsewhere [20]. K562 cells were grown in the same conditions than K562‐A*02:01 cells, as described elsewhere [20].

### 2.2 RNA sequencing

RNA was extracted from K562‐A*02:01 and K562 cell pellets, processed for polyA enrichment, then sequenced by using NEBNext Ultra RNA Library Preparation Kit with random priming. Sequencing was performed using HiSeq 2 × 150 PE HO with the depth of 20 to 25 million reads per sample.

### 2.3 RNA‐informed, Gencode, and inflated reference databases

As Gencode reference transcriptome we used the main annotation Release 33 (GRCh38.p13) [23].

The RNA‐informed database was generated using the RNA sequencing data and by applying a pipeline described elsewhere [20]. Briefly, reads were trimmed using Trim Galore with stringency parameter of 5. Quantification was performed using Salmon (v1.1.0) [24] with decoy‐augmented Gencode v33 human reference transcriptome [23]. In short transcripts, the k‐mer size was reduced to 23 bp and 1000 Gibbs samples were drawn from the posterior distribution of transcript abundances. Only the transcripts that received more than 10 estimated counts in at least one sample were considered to be expressed and their Gencode protein‐coding transcript translation sequences were selected for a common database for MS search.

The inflated reference databases created for testing the impact of database size on Mascot+Percolator performance was generated by reshuffling the entries in Gencode reference database and appending the shuffled entries to the original reference database. To allow increases in size of 25% to 150%, the shuffled protein entries were cut off at the corresponding percentage of their original sequence length.

### 2.4 Datasets

HLA‐I‐bound peptides were isolated from 10^9^ cells from K562‐A*02:01 cell line, through HLA‐I‐peptide elution using W6/32 antibody, as described elsewhere [20].

Tryptic digestions of cell proteome obtained from K562 cell line were carried out as follows: cell pellet was lysed in cell lysis buffer (50 mM HEPES, pH 7.5, 150 mM NaCl, 4% SDS, 2 mM DTT, 0.5% NP40) and heated at 95°C for 10 min. The cell lysate was then diluted to final concentration of 1% SDS with 50 mM HEPES, pH 7.5. Pierce Universal nuclease (Thermofisher scientific) was added according to the manufacturer's recommendations and incubated at 37°C for 30 min under shaking condition (300 rpm). Protein concentration was determined using Pierce BCA protein assay kit (Thermofisher scientific) and 50 μg of protein was used for proteome digestion. Proteins were reduced with 5 mM DTT for 30 min at 37°C and alkylated by the addition of 20 mM iodoacetamide and incubation for 30 min at room temperature in the dark. The reaction was quenched by incubation with 20 mM DTT for 15 min at room temperature before purification with SP3 beads [25], and elution for proteome digestion with trypsin (Promega) at protease to proteome weight ratio of 1:25 at 37°C for 16 h.

The synthetic peptide library contained 9, 10, or 15 amino acid long peptides (*n* = 2981 unique peptide sequences and 4147 PSMs) related to CD4^+^ and CD8^+^ T cell response to Dengue and VZV viruses. The Dengue and VZV synthetic peptides utilized in this study were selected for analysis because they were already available in‐house and synthesized for separate epitope identification studies [26]. The selection and characterization of these peptides has been described previously [27, 28, 29, 30, 31, 32, 33, 34]. Each of the peptides in synthetic peptide libraries was derived from respective Dengue and VZV proteomes. Peptides were originally selected for other studies based on bioinformatic analyses of predicted capacity to bind various common HLA‐I and ‐II alleles in the general worldwide population. The set of Dengue protein sequences of provenance represent all four Dengue serotypes and several different variant isolates. The VZV peptides were primarily derived from the attenuated varicella vaccine strain vOka and a few variant isolates. Peptides were grouped in four library batches, with each peptide measured at the concentration of 0.0625 pmol/μl. For each pool, 8 μl was injected in the instrument, thereby measuring 500 fmol of each peptide.

### 2.5 Mass spectrometry

MS data of HLA‐I immunopeptidomes were originally collected using either Orbitrap Fusion Lumos mass spectrometer coupled to an Ultimate 3000 RSLC nano pump (both from ThermoFisherScientific), as described elsewhere [20]. The same method and instrument were used for the synthetic peptide library measurement. MS data of tryptic digestions of cell proteome were measured through Thermo Scientific Orbitrap Exploris 480 mass spectrometer. Digested proteome samples were injected using an Ultimate 3000 RSLC nano pump (both from ThermoFisherScientific). Briefly, 0.5 μg of each sample was loaded and separated by a nanoflow HPLC (RSLC Ultimate 3000) on an Easy‐spray C18 nano column (30 cm length, 75 μm internal diameter). Peptides were eluted with a linear gradient of 5% to 45% buffer B (80% ACN, 0.1% formic acid) at a flow rate of 300 nl/min over 58 min at 50°C. The instrument was programmed within Xcalibur 3.1.66.10 to acquire MS data in a Data Dependent Acquisition mode using Top 30 precursor ions. We acquired one full‐scan MS spectrum at a resolution of 60,000 with a normalized automatic gain control (AGC) target value of 300% and a scan range of 350 to 1600 m/z. The MS2 fragmentation was conducted using HCD collision energy (28%) with an orbitrap resolution of 15,000. The normalized AGC target value was set up at 100% with a max injection time of 40 ms. A dynamic exclusion of 22 s and 2 to 6 included charged states were defined within this method.

### 2.6 iBench implementation for construction of ground truth datasets

The iBench software package is written in Python. iBench uses as input MS files (mgf or mzML format) and generates constructed ground truth datasets mapping scan numbers to identified peptides, an artificial reference database, and a reindexed mgf or mzML file with the scans of interest selected (Figure 1A). iBench can be combined with the main database search engines such as MaxQuant, Mascot, and PEAKS, as well as Percolator identifications (Figure 1B). A constructed ground truth dataset can be provided from MS files derived from tryptic digestions of proteomes, HLA‐I immunopeptidomes, or synthetic peptide libraries (see Table 1 for details on the benefits of each). With default settings, iBench filters PSMs identified in those experimental datasets to (i) provide a single peptide per MS2 spectrum (no chimeric spectra allowed), (ii) remove I/L redundant peptides, (iii) remove peptides identified with PTMs. The removal of modified peptides is to ensure that only peptides with the highest confidence are assigned. However, if the users were eager to analyze the impact of PTMs, they can set the flag “filterPTMs” to “False” in the iBench config file. In such cases, the user should be aware of the greater risks of misassignment and search engine specific biases in identification. iBench also starts the creation of the constructed reference database by removing all sequences included in the constructed ground truth datasets from the original reference database, replacing all occurrences of the peptides in the original reference database with randomly sampled peptide sequences.

The peptides are then embedded in the constructed reference database according to the frequency of each peptide stratum selected by the user. In the current version of iBench, the possible strata are canonical non‐spliced, *cis*‐spliced and *trans*‐spliced peptides, although further strata could be added in future developments of iBench. For a canonical peptide, the complete sequence is embedded in a random location in the proteome FASTA file (along with a preceding K residue for tryptic datasets). For a *cis*‐spliced peptide, a random point in the sequence is selected as a splice site and the peptide is split into two subsequences at that point. The subsequences are embedded in a randomly selected protein with 1 to 25 randomly selected amino acid residues added between them. In the case of a *trans*‐spliced peptide, iBench splits the peptide into two subsequences and the sub sequences are embedded in two different randomly selected proteins. Therefore, the latter are peptides that cannot be generated by either peptide hydrolysis or *cis*‐peptide splicing of canonical proteins.

Once, these sequences have been added, iBench iterates to ensure that no *trans*‐spliced peptides are present in the constructed reference database as canonical or *cis*‐spliced peptides, and no *cis*‐spliced peptides are present as canonical peptides.

Depending on the peptide identification method tested via iBench, both *cis*‐ and *trans*‐spliced peptides could be used as unidentifiable peptides, that is, peptide sequences that cannot be identified by the tested method. In the present study, we used only *trans*‐spliced peptides as unidentifiable peptides and had no *cis*‐spliced peptides in the constructed ground truth PSM datasets and cognate database. In this use case, iBench did not perform the validation that *trans*‐spliced peptides were not also possible via peptide *cis*‐splicing, since both strata were unidentifiable by the search engine used in our representative analysis, that is, a standard Mascot+Percolator.

iBench also reads in the mzML or mgf files provided and returns a single mzML or mgf file containing reindexed scan numbers. The user can then apply the chosen identification method(s) to the reindexed file using the constructed reference database.

### 2.7 Identifications of PSMs for the ground truth datasets in this study (iBench input generation)

To generate a constructed ground truth PSM dataset for the trypsin proteome digestions and HLA‐I immunopeptidomes, we searched the original MS datasets with Mascot against an RNA‐informed database generated as described elsewhere [20]. In terms of search settings, we set enzyme specificities to ‘trypsin’ for the trypsin proteomes and ‘non‐specific’ for the HLA‐I immunopeptidomes. Precursor mass tolerances were set to 5 ppm and fragment ion mass tolerances were set to 0.02 Da. We then applied Percolator using engine score, delta score, charge, sequence length, ms1 error and absolute ms1 error as features. The PSMs with q‐value less than 0.01 were selected as ground truth identifications. The tryptic proteome digestions provided a larger number of PSMs identified at 1% FDR and we wanted to compare the peptide identifications in a tryptic proteome digestion and a HLA‐I immunopeptidome on equal footing. Therefore, we sampled the tryptic proteome digestion dataset so that both constructed ground truth datasets contained similar numbers of PSMs and peptides (1689 unique peptides for the HLA‐I immunopeptidome and 1829 unique peptides for the tryptic proteome digestion).

In the case of the synthetic peptide library, the MS data for each of the four raw files were searched with PEAKS DB and MaxQuant against a list of the synthetic peptides which were used in each batch. Percolator was applied to the MaxQuant search results with the same feature set used for Mascot. The PEAKS DB results were exported at 1% FDR. These results were used as input for iBench, which selected only unique peptides with PSMs identified by both PEAKS DB and MaxQuant+Percolator at 1% FDR leading to 3429 PSMs in the constructed ground truth dataset.

### 2.8 iBench benchmarking framework

For the benchmark plots provided, all PR and ROC curves were generated using the iBench analysis functionality (see Result section; a more detailed description is available in the GitHub repository mentioned in the Associated Data and Software section). The iBench analysis was run separately on each set of identifications and then all PR curves for each sample type were replotted on the same axis using Python and the Plotly library so that performance could be compared.

### 2.9 FDR estimation by applying Mascot ± Percolator and iBench

As with the benchmarking framework, the true versus estimated FDR plots were generated using the iBench analysis functionality and replotted using Python and the Plotly library. For each point estimate the true FDR was calculated by considering all target PSMs with q‐value less than that value and comparing the number of correct PSMs (C) and incorrect PSMs (I) to estimate the FDR as $FDR=100\frac{I}{C+I}$.

### 2.10 MS2 spectra characteristics

For each MS2 spectrum in our ground truth datasets, iBench can compute MS2 spectra characteristics. The default features are reported in Table 2.

**TABLE 2:** MS2 spectrum and peptide features included in iBench

Feature	Description
charge	The charge of the peptide.
sequenceLength	The length of the peptide.
hydrophobicityIndex	The hydrophobicity index of the peptide.
mass	The mass of the peptide.
ms2Coverage	The number of fragmentation positions on the peptide backbone for which there is at least one mz matched peak in the MS2 spectrum divided by the total number of possible fragmentation positions.
signalToNoise	The ratio of the intensities of the matched peaks to the unmatched peaks in the MS2 spectra.

### 2.11 Statistical analysis

All statistical analysis has been implemented in Python. All statistics for performance measurement are described in the benchmarking framework. FDR calculation is described in the respective methods sections.

## 3 RESULTS

### 3.1 Overview of iBench

The Python based iBench software package consists of two parts: (i) construction of ground truth dataset, and (ii) performance evaluation (Figure 1A).

The ground truth dataset (i) consists of a constructed MS dataset and a constructed reference database. First, user‐provided annotated MS2 spectra are filtered, selected and combined into a constructed ground truth dataset. MS2 spectra can be derived from various sources, such as tryptic digestions of proteomes, HLA‐I immunopeptidomes, or synthetic peptide libraries (see Table 1 for details on the benefits of each). iBench allows for flexible input formats for both MS data (mgf or mzML) and MS search engines such as MaxQuant, Mascot, and PEAKS, as well as combinations with Percolator (Figure 1B). Along this process, iBench calculates a number of peptide features such as hydrophobicity index as well as features describing the match to the MS2 spectrum such as signal to noise ratio (Table 2, Figure 1A). The default feature list present in iBench could be easily modified by the user by adding new customized features. These features can later be investigated for their impact on the MS identification method.

The assigned peptide sequences of the constructed ground truth dataset build the basis to generate the respective database for benchmarking. Starting from a user‐provided reference database, which should reflect the complexity of the desired analysis in terms of number of database entries and entry length distribution, all assigned peptide sequences of the constructed ground truth dataset are encoded as defined by the user. In the current version of iBench, each peptide can be categorized as either canonical non‐spliced, *cis*‐spliced, or *trans*‐spliced. Once the benchmarking database is constructed, iBench provides the user with both, the constructed ground truth dataset with known annotations as well as the constructed database in FASTA format. These iBench outputs can then be used by the user as inputs for their search algorithms under investigation.

In this study, we demonstrate the functionality of iBench by benchmarking the performance of Mascot+Percolator on constructed ground truth datasets derived from tryptic proteome digestions, HLA‐I immunopeptidomes, and synthetic peptide libraries. In the examples shown here, we applied iBench to MS search strategies that could identify only canonical peptides, namely ‘identifiable’ peptides. Therefore, both *cis*‐ and *trans*‐spliced would have been ‘unidentifiable’ peptides. For the sake of simplicity, during the construction of the constructed ground truth databases in this study, only spliced peptides were included and hence were considered as ‘unidentifiable’ peptides.

After the user has applied their identification method (or multiple identification methods) to the constructed ground truth datasets, iBench continues with performance evaluation (ii) via standard benchmarking tests, producing various statistical outputs (Figure 1B, Table 3). In the first step of this process, iBench creates a “query table” merging the constructed datasets (the ground truth peptides and their reindexed scan numbers along with iBench calculated PSM features) with the user's identifications and the score assigned to each PSM. This query table is used to benchmark the method(s), which the user applied. PR and ROC curves are generated by calculating correct and incorrect identifications above a scoring threshold, which is varied between the minimum search engine score and the maximum search engine score. True FDR at a given cut off can be identified by calculating the fraction of incorrectly assigned PSMs above that threshold. If the user provides the decoy identifications of the search algorithm under investigation, iBench produces graphical outputs comparing search engine score distributions of incorrect target PSMs against decoy PSMs, particularly with respect to possible confounding variables such as sequence length (Figure 1B).

**TABLE 3:** Benchmarking outputs included in iBench

Output	Description
Precision‐Recall (PR) Curve	Plot of precision against recall when we vary the threshold from the minimum score assigned to the maximum score assigned.
Receiver‐Operator Characteristic (ROC) Curve	Plot of true positive rate against false positive rate when we vary the threshold from the minimum score assigned to the maximum score assigned.
Feature Distributions	Scatter plots and violin plots as appropriate for the feature for the distribution of search engine score against the feature values for correct and incorrect PSMs

These iBench generated benchmarking outputs provide insight into the performance of a search algorithm (PR curves, ROC curves, etc.) and potential sources of bias in the identification such as scatter plots and violin plots of search engine scores against confounding variables for correct, incorrect, and decoy PSMs. An example of the iBench output is provided in the supplemental data which shows all of the benchmarking and analysis plots (File S1). This example also provides a useful practical example of using iBench to compare different identification methods. For the purposes of the example, we benchmarked Percolator rescoring of Mascot search results using three slightly different feature sets, although the same iBench analysis could be generated for many diverse identification tools. The PR and ROC curves provide an easy comparison of identification performance, while analysis of FDR and confounding variable distributions could help to avoid bias or inaccurate FDR estimation of a method analyzed. The example output can also be generated by downloading the repository from GitHub and following the instructions in the README (see Associated Data and Software section).

### 3.2 Mascot+Percolator performance in constructed ground truth datasets of tryptic proteome digestions and HLA‐I immunopeptidomes with a variable frequency of identifiable canonical peptides

As a first example of an iBench benchmarking strategy using constructed ground truth datasets and cognate databases, we applied iBench to tryptic proteome digestions. The selection of the PSMs used by iBench to construct the ground truth dataset is described in the Materials and Methods. As a proof of concept, by applying iBench we constructed seven reference databases, which contained only a portion (between 30% and 90%) of the peptides present in the constructed ground truth dataset. Therefore, only 30% to 90% of the peptides present in the constructed ground truth dataset were identifiable by the database‐dependent search engine. We then applied Mascot+Percolator on this constructed ground truth dataset using the seven constructed reference databases. Thereby, we could estimate the impact of any kind of noncanonical peptides, which are not identifiable through a standard database‐dependent Mascot+Percolator search, on the identification of canonical peptides. We hypothesized that the search engine performance in the identification of canonical peptides would vary from an optimal performance‐, that is, whilst using a constructed reference database that had no unidentifiable peptides – to less performant behaviors whilst using constructed databases with a prevalence of unidentifiable peptides.

**FIGURE 2:**
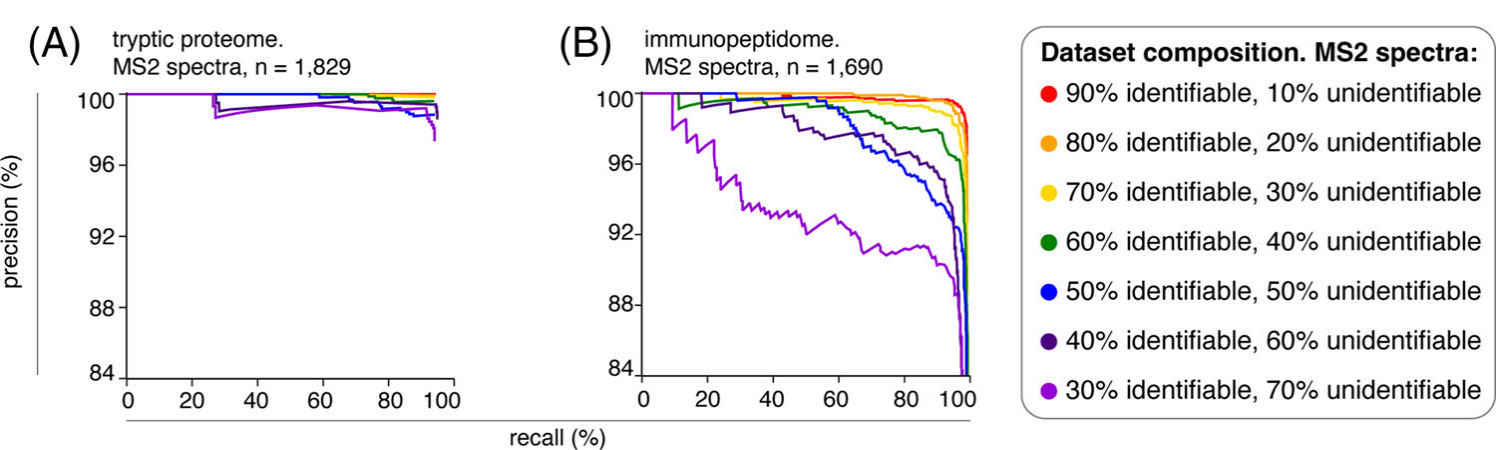
Canonical peptide identification performances of Mascot+Percolator in ground truth datasets constructed from tryptic proteome digestions and HLA‐I immunopeptidomes varying the frequency of identifiable peptides. (A) PR curves for the identification of canonical peptides by Mascot+Percolator in ground truth datasets constructed from tryptic proteome digestions of K562 cell line. (B) PR curves for the identification of canonical peptides by Mascot+Percolator in ground truth datasets constructed from HLA‐I immunopeptidomes of K562‐A*02:01. Each curve represents a different frequency of canonical peptides present in the ground truth datasets that can be identified using the cognate ground truth reference database, and is generated by applying a range of scoring cut‐offs. All datasets were obtained through measurement by Orbitrap Fusion Lumos. The number of PSM in each constructed ground truth dataset is reported.

To test this, we computed PR curves for canonical peptide identification of Mascot+Percolator search engine by applying iBench. The precision in the identification of canonical peptides was defined as number of correctly identified canonical peptides over number of identified canonical peptides. Therefore, the denominator included both canonical peptides identified as such and unidentifiable peptides wrongly assigned with one of the sequences of the canonical peptides by Mascot+Percolator. The larger the pool of peptides misassigned as canonical peptides, the lower is the precision in the identification of canonical peptides. Optimal methods have the highest precision at the highest recall. In this analysis, the increase of the unidentifiable peptide pool in the constructed ground truth dataset had only a slight impact on the PR curve in the tryptic proteome digestions. Indeed, while the dataset containing 10% unidentifiable peptides achieved essentially perfect performance (100% precision at any cut off), even the datasets with 70% unidentifiable peptides showed strong performance (Figure 2A).

We then repeated the benchmarking strategy using constructed ground truth datasets and cognate reference databases by applying iBench to K562‐A*02:01 HLA‐I immunopeptidomes. The selection of the PSMs used by iBench is described in the Materials and Methods. As done for the tryptic proteome digestions, we varied the ratio of peptides present in this constructed ground truth dataset that could be identified by using one of the seven constructed reference databases. We then applied Mascot‐Percolator constructed ground truth dataset. Again, in constructing the ground truth HLA‐I immunopeptidome datasets, Mascot+Percolator was used to select high confidence PSMs of canonical peptides. Thereby, we could measure the search engine performance in the identification of canonical peptides in a HLA‐I immunopeptidome‐derived dataset that contained different proportions of identifiable (canonical) peptides and unidentifiable (noncanonical) peptides. In this example, the increase of the unidentifiable peptide pool in the constructed ground truth dataset affected the PR curves (Figure 2B), in a much stronger manner than in the constructed ground truth dataset of tryptic proteome digestions (Figure 2A). Performance across all constructed ground truth datasets was reduced as compared to the tryptic proteome digestions, and the variation of the pool of unidentifiable peptides in the constructed reference dataset more remarkably affected the method performance. For instance, when using a constructed reference database that could allow the identification of 30% of the peptides in the constructed ground truth dataset, Mascot‐Percolator had a precision of 91% with a recall of 80%. Conversely, when using a constructed reference database that could allow the identification of 90% of the peptides in the constructed ground truth dataset, Mascot‐Percolator had the same recall but an improvement in precision up to 99% (Figure 2B).

### 3.3 Mascot+Percolator performance in ground truth constructed datasets of tryptic proteome digestions and HLA‐I immunopeptidomes using ground truth reference databases of varying size

In our first analysis (Figure 2), we leveraged iBench to test the impact of peptides present in the constructed datasets but not in the constructed reference database. We illustrated that increasing the percentage of unidentifiable peptides using a given constructed reference database had a negative impact on the identification of the identifiable peptides. Our next goal was to test the opposite case. By applying iBench, we constructed seven reference databases that could allow the identification of a fixed number of peptides present in the ground truth dataset, and had a variable database size. The larger the database, the larger the pool of identifiable peptides not present in the dataset. This test allowed us to measure the impact of the reference database size on the search engine performance. Recent strategies of limiting the size of the reference database through RNA‐sequencing information have been proposed to boost MS search engine performance [35] and other strategies have been proposed to address the frequently discussed problems of working in enlarged search spaces [11].

**FIGURE 3:**
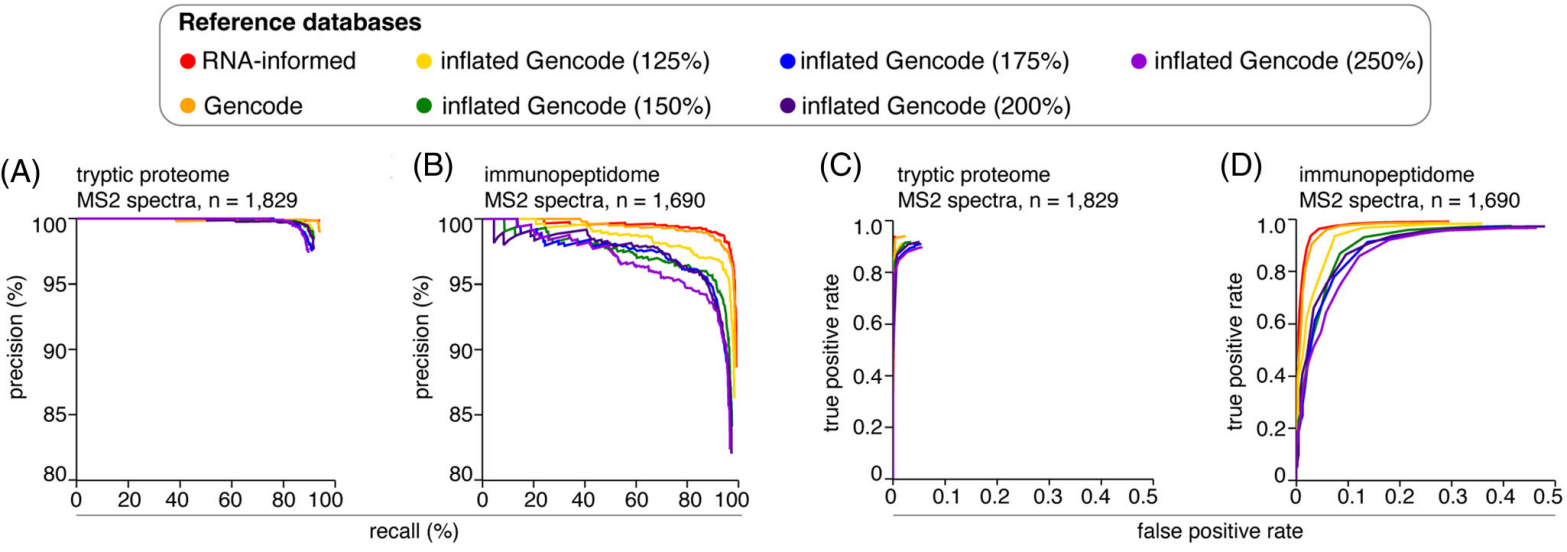
Canonical peptide identification performances of Mascot+Percolator in ground truth datasets constructed from tryptic proteome digestions and HLA‐I immunopeptidomes using cognate databases of various sizes. (A) PR curves for the identification of canonical peptides by Mascot+Percolator in ground truth datasets constructed from tryptic proteome digestions of K562 cell line. (B) PR curves for the identification of canonical peptides by Mascot+Percolator in ground truth datasets constructed from HLA‐I immunopeptidomes of K562‐A*02:01. (C) ROC curves for the identification of canonical peptides by Mascot+Percolator in ground truth datasets constructed from tryptic proteome digestions of K562 cell line. (D) ROC curves for the identification of canonical peptides by Mascot+Percolator in ground truth datasets constructed from HLA‐I immunopeptidomes of K562‐A*02:01. Each PR or ROC curves represent identifications using reference databases of differing size, and are generated by applying a range of scoring cut‐offs. All datasets were obtained through measurement by Orbitrap Exploris 480. The number of PSMs in each constructed ground truth dataset is reported.

To address this subject matter, by applying iBench we created seven reference databases of varying sizes to be used by Mascot+Percolator on the constructed ground truth dataset derived from K562 tryptic proteome digestions and K562‐A*02:01 HLA‐I immunopeptidomes. All ground truth databases were created so that 70% of peptides in the constructed ground truth datasets were identifiable and 30% unidentifiable. By applying iBench, we created ground truth databases using (i) an RNA‐informed reference database, (ii) the Gencode reference database, and (iii) progressively enlarged reference databases (up to 250% of the size of the original Gencode database). The enlarged reference databases were created by appending randomized copies to the Gencode database (see Materials and Methods for more details). We calculated PR curves for both kinds of constructed ground truth datasets (Figure 3A,B). In this case, since the percentage of identifiable peptides was set constant between datasets by iBench, we also calculated ROC curves for both datasets (Figure 3C,D). ROC curves which report true positive rate (equivalent to recall) i.e., number of correctly identified peptides divided by the number of all identifiable peptides in the dataset – on the Y‐axis, and false positive rate i.e., number of incorrectly identified peptides divided by the percentage of unidentifiable peptides in the dataset – on the X‐axis.

For the constructed ground truth datasets derived from tryptic proteome digestions, we observed a near perfect performance as measured by both PR and ROC curves when using a constructed reference database of the same size as the original RNA‐informed database (Figure 3A,C). Even with recall or true positive rate above 90%, precision remained above 99% and the false positive rate did not exceed 1%, even at the lowest scoring threshold. Performance remained reasonably robust while searching in the constructed tryptic proteome digestion datasets even when using the Gencode and inflated reference databases. In all cases, the true positive rate exceeded 80% before the false positive rate exceeded 1% (Figure 3C).

In contrast, the analysis of the constructed ground truth dataset derived from the HLA‐I immunopeptidomes was more sensitive to the increase in cognate reference database size. As with the tryptic proteome digestion dataset, optimal performance was achieved when ground truth database construction was based on the RNA‐informed reference database. However, in this case there was a greater risk of peptide sequence misassignment compared to the tryptic proteome digestion dataset, with a false positive rate of 29% for the lowest scoring threshold (Figure 3D). The impact of increasing reference database size was also more remarkable in the constructed ground truth HLA‐I immunopeptidome dataset than the tryptic proteome digestions. Indeed, we observed performance quickly decayed on both PR and ROC curves when using the larger constructed reference databases. When Mascot+Percolator used constructed reference databases with a size of 200% to 250% compared to the original Gencode reference database, the precision dropped below 99% at recall below 25% (Figure 3B), and the false positive rate exceeded 1% even when the true positive rate was below 40% (Figure 3D). When the largest constructed reference databases were used, the false positive rate reached close to 50% for the lowest scoring thresholds, thus illustrating the dangers of working with such enlarged databases (Figure 3D).

### 3.4 Experimentally measured FDRs of Mascot+Percolator performance computed in constructed ground truth datasets of synthetic peptide libraries

The strategy adopted so far constructed ground truth datasets using high confidence PSMs identified by a given search engine (Mascot+Percolator, in this case) in the same sample type wherein we wanted to test the search engine performance. Through this strategy iBench could efficiently estimate, for instance, the impact of the dataset structure and enzyme specificity (Table 1, Figure 2). Nonetheless, the PSMs selected by iBench to construct the ground truth datasets were identified by the same search engine with high confidence in the original datasets. This raises two issues; firstly, the PSMs included in the constructed ground truth datasets were already identified under the same conditions and by the same method, so these were likely MS2 spectra which the search engine already well identified. Secondly, we could not have full confidence in the PSMs used to construct the ground truth datasets because they were initially identified in a dataset of unknown peptide content. For instance, if a search engine severely underestimated the FDR but consistently identified the same incorrect PSMs between the original dataset and the constructed ground truth dataset, then iBench could wrongly estimate the methods‘ precision.

If we wanted to measure the experimental FDR of a given search engine, for example, we could adopt a different strategy. Rather than using the same kind of sample both as a source for the constructed ground truth dataset and the final search engine application, we could generate a constructed ground truth dataset using PSMs with a range of MS2 spectral quality identified in a synthetic peptide library by applying iBench. As a proof‐of‐principle, we adopted this strategy using four MS files of synthetic peptide libraries derived from 9, 10, and 15 amino acid long peptides previously investigated as potential pathogen epitopes (see Materials and Methods section). We also used PEAKS DB and MaxQuant in the initial identification of these peptides to ensure that the search engine used in benchmarking (Mascot+Percolator) was not used in the ground truth dataset construction (see Materials and Methods section).

**FIGURE 4:**
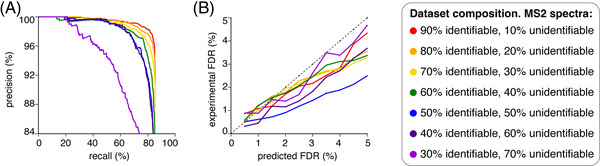
Estimated and measured FDR of Mascot+Percolator in ground truth datasets constructed from synthetic peptide libraries. (A) PR curves for the identification of canonical peptides by Mascot+Percolator in ground truth datasets derived from synthetic peptide libraries. Datasets were obtained through measurement by Orbitrap Fusion Lumos. Curves represent the performances by applying a range of scoring cut‐offs. Each curve represents a different frequency of canonical peptides present in the ground truth dataset that can be identified using the cognate ground truth reference database. The constructed ground truth dataset contained 3429 MS2 spectra. (B) Plot of the experimentally observed FDRs against the corresponding q‐value cut off as estimated by Percolator on the constructed ground truth datasets derived from synthetic peptide libraries. Dots represent the observed FDR at q‐value cut‐offs from 0.5% to 5%. The dashed line represents a theoretical perfect FDR prediction.

This benchmarking strategy resulted in a greater challenge to the search engine, with the observed precision much lower at corresponding recall values than for the previously constructed datasets (Figure 4A and Figure 2B could be compared). Even with 90% of PSMs identifiable in the constructed ground truth dataset using the cognate reference database, a recall of 65% was achieved at 99% precision (Figure 4A), compared to recalls in excess of 90% at 99% precision for the constructed ground truth datasets derived from HLA‐I immunopeptidomes shown in Figure 2B. Using a ground truth dataset constructed on a synthetic peptide library, the maximum recall observed was also lower than when using a ground truth dataset constructed on an HLA‐I immunopeptidomes (84% in Figure 4A vs. 99% in Figure 2B). Despite this difference in the search engine performance, the percentage of peptides, present in the ground truth datasets constructed using either the HLA‐I immunopeptidomes (Figure 2B) or a synthetic peptide library (Figure 4A), that could be identified using the different constructed reference databases had a remarkable impact on search engine performance.

In addition, by applying iBench on a synthetic peptide library dataset, we had a much greater confidence in the PSMs included in the constructed ground truth dataset since we knew what synthetic peptides were present in the library. This could allow iBench to assess the FDR prediction of a given search engine, Mascot+Percolator in our representative study. In Figure 4B, we presented the experimental FDR i.e., the percentage of wrongly assigned PSMs by Mascot+Percolator – observed at various q‐value/FDR cut offs. Percolator was applied using target‐decoy competition for FDR estimation. In this case there was no obvious impact of the percentage of unidentifiable peptides in the constructed ground truth dataset on the FDR estimated by Mascot+Percolator. In most cases the FDR prediction of Mascot‐Percolator was conservative, and the predicted FDR was greater than the experimentally measured FDR, although some underestimation of predicted FDR by Mascot+Percolator emerged (Figure 4B).

### 3.5 Practical aspects of iBench application

Regarding the informatic aspects, iBench software supports Linux, Mac OS, and Windows operating systems. The software requires conda for installation and can be executed via command line. Due to the complexity of the reference database creation, iBench does not allow parallel execution and so runs on one core only.

Regarding the computational time required by iBench, as representative estimation, we computed the time taken to create the ground truth reference database (“createDB” pipeline) for varying the reference database size (Table 4) and the number of high confidence peptides (Table 5). For all benchmarking runs we embedded 70% canonical peptides and 30% unidentifiable peptides to match the settings used in our investigation varying the database size (Figure 3). We observed that iBench execution time was not heavily impacted by the size of the reference database (Table 4), with an execution time of less than 10 min even when using a reference database 2.5 times larger than the Gencode database. The number of high confidence peptides embedded in the proteome had a much greater impact on the execution time (Table 5). However, even for the most extreme case, where 20,000 high confidence peptides were embedded in the proteome, the execution time was less than 80 min, which we feel is perfectly acceptable given that the same reference database can then be used to benchmark multiple identification methods. We also note that given that iBench runs on one core only and is not highly memory intensive, the user can easily leave reference database creation running as a background task without fear of over‐allocation of computing resources.

**TABLE 4:** Run times observed for iBench “createDB” pipeline for increasing Database size

Number of peptides	Database size (Entries)	Database size (Residues)	Mean protein length	Run time (Minutes)
5000	43,578	17,401,687	399	5.54
5000	100,551	38,113,847	379	6.14
5000	201,102	57,145,643	284	7.66
5000	201,102	76,227,694	379	7.91
5000	301,653	95,259,490	315	8.90

This table provides the run time in minutes on an Apple M1 CPU running the iBench “createDB” pipeline embedding 10,000 peptides into databases of increasing size.

**TABLE 5:** Run times observed for iBench “createDB” pipeline for varying number of high confidence peptides

Number of peptides	Database size (Entries)	Database size (Residues)	Mean protein length	Run time (Minutes)
1000	100,551	38,113,847	379	1.31
2000	100,551	38,113,847	379	1.84
5000	100,551	38,113,847	379	6.14
10,000	100,551	38,113,847	379	21.49
20,000	100,551	38,113,847	379	79.42

This table provides the run time in minutes on an Apple M1 CPU running the iBench “createDB” pipeline embedding an increasing number peptides into the Gencode v.33 database.

## 4 DISCUSSION

iBench is a useful open‐source software, which can apply a standardized evaluation of search engine performances. The software can accept input from Mascot, PEAKS, and MaxQuant search engines as well as Percolator output for both ground truth dataset construction and benchmarking analysis. The software can provide insights into the performance of an identification method/strategy in terms of specificity and sensitivity (corresponding to precision and recall) as well as the accuracy of the method's FDR prediction and possible sources of bias. Although in this study we focused on methods that could identify only canonical peptides, iBench could also be used to benchmark novel methods able to identify noncanonical *cis*‐spliced peptides. For instance, a ‘manual’ version of iBench was used to benchmark database search engine performance in the identification of non‐spliced and *cis*‐spliced peptides in HLA‐I immunopeptidomes, and only *trans*‐spliced peptides were used as ‘unidentifiable’ peptides in constructed ground truth datasets and cognate databases [20]. Since iBench is an open‐source software, we do not exclude that it could be developed to generate also other peptide strata (e.g., scrambled peptides); then, iBench could be coupled to search engines able to identify also *trans*‐spliced peptides, thereby allowing the evaluation of the method performance in identifying also this kind of noncanonical peptides. The software could be expanded even further, for example, by embedding reverse‐translated sequences into modified RNA‐informed databases, which could allow for benchmarking of methods designed to identify cryptic peptides using various RNA‐sequencing based strategies [3].

As a proof of concept, in the results shown in Figure 2 and Figure 3, we tested Mascot+Percolator on ground truth datasets that were constructed using high confidence PSMs identified by a standard Mascot+Percolator pipeline. Therefore, our analysis focused exclusively on the impact of unidentifiable peptides and reference database size on the search engine and rescoring performance in the identification of canonical peptides. By constructing ground truth datasets using original datasets that have the same characteristic as the datasets that the user aims to analyze by the search engine(s), the user has the advantage of testing the search engine performance on the peptides with features similar to the target datasets. This issue is well represented by the proof‐of‐concept benchmarking that we proposed in this manuscript. Indeed, tryptic proteome digestions and HLA‐I immunopeptidomes differ in many aspects. According to the iBench‐mediated benchmarking, Mascot+Percolator performance was differently affected by a variation of the frequency of unidentifiable peptides and the size of the reference database in the two types of datasets, with a much greater impact shown for the HLA‐immunopeptidome datasets. This illustrates the importance of having a well‐informed reference database, maximizing the percentage of identifiable peptides while minimizing database size. While these considerations were not remarkable in the analysis of the tryptic proteome digestions, they were critical in exploring the murky waters of HLA‐I immunopeptidomics.

In the case of the ground truth datasets constructed on tryptic proteome digestions, the Mascot+Percolator performance was relatively robust to decreasing the percentage of identifiable peptides in the ground truth datasets, with precision never dropping below 97% even for the lowest percentage of identifiable peptides at the lowest scoring cut off. The performance was much more variable in the ground truth datasets constructed on HLA‐I immunopeptidome dataset, particularly when less than 70% of peptides present in the constructed ground truth datasets were identifiable using the constructed reference database. Similarly, we observed a stronger impact of the reference database size on Mascot+Percolator when applied to constructed ground truth datasets derived from HLA‐I immunopeptidomes than tryptic proteome digestion.

Our proof‐of‐concept application of iBench also demonstrated how the use of ground truth datasets constructed on synthetic peptide libraries can provide a more challenging task for the search engine, and an estimation of the discrepancy between predicted and experimentally measured FDRs.

While the datasets used in our analysis are provided (see Associated Data and Software), the iBench user could also choose from the many publicly available synthetic peptide libraries for the generation of ground truth datasets (*e.g*., [10]). Since iBench allows a selection of the MS2 spectrum and peptide features of the selected PSMs in the constructed ground truth dataset, the user could carefully choose these features to mimic those of the target datasets, wherein the search engine will be finally applied.

Of course, iBench could be used for many different objectives. For example, a search engine A could be used for the ground truth dataset and database construction, and the search engines B, C, and D could be applied to the ground truth constructed datasets for benchmarking. Such a strategy could investigate the specific different performance comparing how well the assignments from search engines B, C, D align with the search engine A. Further information could be gathered by varying the features of the PSMs and cognate peptides assigned to either identifiable or unidentifiable peptide groups, which is one iBench functionality. To note, iBench constructs ground truth datasets and cognate databases using one or more search engines for the selection of the PSMs. This strategy contains a certain level of bias toward the search engine(s) and the parameters used for the construction of the ground truth datasets. This aspect should always be considered in the experimental design and the interpretation of iBench outputs.

### 4.1 Associated data and software

The MS proteomics data have been deposited to the ProteomeXchange Consortium via the PRIDE [36] partner repository with the dataset identifier PXD031709 [20], PXD031812, PXD034056, and PXD034968.

The RNA sequencing data have been deposited in the NCBI Sequence Read Archive database with the accession code PRJNA721129 [20].

The iBench software has been implemented with Python and is available at GitHub (https://pypi.org/project/ibench/).

Analyses were carried out in Python 3.8.

Figures have been generated in Python using the Plotly library and postprocessing was done with Adobe Illustrator v26.2.

MS analysis was carried out with MaxQuant version 1.16.17, Mascot v2.7.01, PEAKS X Pro 10.6. Rescoring was carried out with Percolator version 3.0.5. RAW files were converted to mgf/mzML format for iBench input using ms‐convert GUI (ProteoWizard version 3.0.9134).

## CONFLICT OF INTEREST

The authors declare no conflict of interest.

## Supporting information

Supporting Information
