# Supplementary material for: iBench: A ground truth approach for advanced validation of mass spectrometry identification method
Source: Proteomics. 2022 Oct 17;23(2):2200271. doi: 10.1002/pmic.202200271 (PMC10078205; doi:10.1002/pmic.202200271)

iBench: A ground truth approach for advanced validation of mass spectrometry identification method

John A. Cormican[1], Wai Tuck Soh[1], Michele Mishto[2,3,$], Juliane Liepe[1,$]

1 Max-Planck-Institute for Multidisciplinary Sciences (MPI-NAT), 37077 Göttingen, Germany

2 Centre for Inflammation Biology and Cancer Immunology (CIBCI) & Peter Gorer Department of Immunobiology, King's College London, SE1 1UL London, United Kingdom

3 The Francis Crick Institute, WC2A 3LY London, United Kingdom

[$] Correspondence to: michele.mishto@kcl.ac.uk, jliepe@mpinat.mpg.de.

File S1. A combined PDF document made up of the README file (converted from markdown) for iBench along with an example of iBench output (converted from html). The README provides the user with a detailed description of how to execute iBench with proper configuration and is also available on Github. The example output shows the report provided by iBench in a simple example with 3 different feature sets passed to Percolator. It can also be generated by downloading the iBench code from Github and following the instructions in the README. The file is available at the journal's portal.

iBench

Benchmarking Mass Spectrometry Identification Methods.

iBench is a tool to help you understand the performance of an mass spectrometry identification method

To use iBench you will need:

- A source of ground truth identifications which you are confident in.
- MS data for the ground truth identifications (mgf or mzML format)
- A proteome fasta file to modify.
- An identification method or set of method which you wish to benchmark.

In the example used in this data we have

- Ground Truth Identifications : Synthetic Peptides identified at 1% FDR by both MaxQuant and PEAKS.
- MS data : mgf files for the Synthetic Peptide MS measurments.
- Fasta File : This file comes from the expressed proteome of K562 cell line.
- Identification Method(s) : We provide output files for Mascot search and percolator rescoring for 3 different feature sets.

Set Up

Before Downloading

You will also require conda to use the iBench software.

Setting up your environment:

1) To start with create a new conda environment with python version 3.8:

```
conda create --name ibench python=3.8
```

2) Activate this environment

```
conda activate ibench
```

3) You will then need to install the iBench package:

```
pip install ibench
```

4) To check your installation, run the following command (it is normal for this call to hang for a few seconds on first execution)

```
ibench -h
```

Once you have successfully installed iBench you must run it specifying your pipeline and a config file.

iBench Execution

iBench is executed with two command line arguments --pipeline which specifies the pipeline to be executed.

iBench Pipeline: createDB

The first option for the pipeline is "createDB" which processes the ground truth identifications to produce the ground truth datasets, artificial reference database and reindexed MS files needed to benchmark performance against.

iBench Pipeline: analysis

After running the first pipeline the user should apply their identification method to the reindexed MS files and artificial reference database. These identifications can be provided to iBench for the "analysis" pipeline. This processes the data and produces a report containing multiple figures describing each method's performance.

iBench Config File

The second argument provided to iBench is --config_file. This specifies the location of a yaml file which contains all the meta data needed to run the iBench software. Such data includes locations of search results, original MS data, as well as user preferences on the creation of the database and results output. Full details of the configuration settings possible are given in the table at the bottom of this README and an example config file is provided used in the "Running a Small Example" section.

Running a Small Example

As an example we will demonstrate how iBench could be used to benchmark Mascot with Percolator Rescoring with 3 different feature sets. This example will use ground truth identifications from PEAKS and MaxQuant searches of our synthetic peptide library. Before running this example you will need to download the necessary data with:

```
ibench --pipeline downloadExample
```

This will create a folder called example in your working directory which will allow you to run the iBench example. Alternatively, you could download the required data from figshare.

Create the Ground Truth Dataset

To create the ground truth datasets:

```
ibench --config_file example/config.yml --pipeline createDB
```

Next Steps

To provide a working example we have performed a Mascot search on the output data and provided

Running the Analysis

You will now

```
ibench --config_file example/config.yml --pipeline analysis
```

This will create a html report with all relevant plots to compare the performance of the 3 pipelines.

Full List of iBench Config Settings

This provides all of the configuration setting both optional and required for each pipeline.

Required for any Execution

iBench will always require an identifier for your experiment and an output folder.

Key	Description
identifier	Some identifier or experiment title for your iBench analysis.
outputFolder	The folder into which all iBench outputs will be written.

Required for createDB Execution

The following config settings can be used

Key	Description
scanFolder	A folder containing all of the MS files.
scanFormat	The format of the MS files (either mgf or mzML).
searchResults	A list of all of the search results used. The search results should be a list of outputs from PEAKS, MaxQuant, Mascot, or Percolator, see more details below.
canonicalFraction	The fraction of the ground truth identifications which should be embedded as canonical sequences in the artificial reference.
cissplicedFraction	The fraction of the ground truth identifications which should be embedded as cisspliced sequences in the artificial reference.
transsplicedFraction	The fraction of the ground truth identifications which should be transspliced or trapping sequences in the artificial reference (not discoverable as canonical or cisspliced).
ms2Accuracy	The m/z accuracy on the measurement of the MS2 spectrum (needed for calculating coverage and signal to noise features).
enzyme	The enzyme used to produce the peptides, can be set to trypsin, otherwise the default value is None (unspecific digestion).
proteome	The location of a proteome fasta file which will be modified to produce the artificial reference database.

Optional for createDB Execution

The following config settings can be used for specific user requirements in the createDB pipeline.

Key	Description
closenessCutOff	The minimum number of residues difference between the shorter of any pair of peptides and their longest shared subsequence (default is 1 if no cisspliced peptides are required and 3 if cisspliced peptides are being used.
randomSeed	The random seed to use to ensure reproducibility of iBench execution (default=42).
maxSequenceLength	Define the maximum sequence length of peptides to be used in the ground truth

Key	Description
	identifications.
minSequenceLength	Define the minimum sequence length of peptides to be used in the ground truth identifications.
filterPTMs	Whether to filter modified peptides from the ground truth identificaitons, by default this will be True to ensure maximum confidence in the ground truth dataset, however it can be set to False if the user wishes to investigate PTMs.

Required for analysis Execution

The following config settings can be used

Key	Description
scanFolder	A folder containing all of the MS files.
scanFormat	The format of the MS files (either mgf or mzML).
benchmarkResults	A list of all of the search results to benchmark. The search results should be a list of outputs from PEAKS, MaxQuant, Mascot, or Percolator, see more details below.

Specifying Search Results

When providing search results, iBench requires more information than a location. To see how this should be formated in yaml see the example/config.yml file.

For ground truth identifications you will require:

Key	Description
searchEngine	mascot, peaks, percolator, or maxquant
resultsLocation	The location of the results file.
scoreLimit	The score above which we should consider PSMs for ground truth identifications.
qValueLimit	Only for mascot or percolator. The q-value below which we should consider PSMs for ground truth identifications.
identificationGroup	If you are using multiple search engines for the original search and only wish to use PSMs identified by both, assign them to the same identificationGroup. Otherwise ensure that all results belong to separate identification groups.

For benchmarking results you will require:

Key	Description
name	The name of your identification method.
searchEngine	mascot, peaks, percolator, or maxquant.
resultsLocation	The location of the results file.

Key	Description
decoyLocation	(optional) The location of your decoy PSMs if using Percolator or Mascot.
colour	The colour you want to identify the method with in the iBench output plots. Can be any CSS colour.

iBench Performance Report for Example

Precision-Recall Curves

This plots the precision (correct identifications divided by all identifications) against the recall (true identifications divided by possible identifications) for each identification method as we vary the scoring the threshold from the maximum to the minimum score in the data.

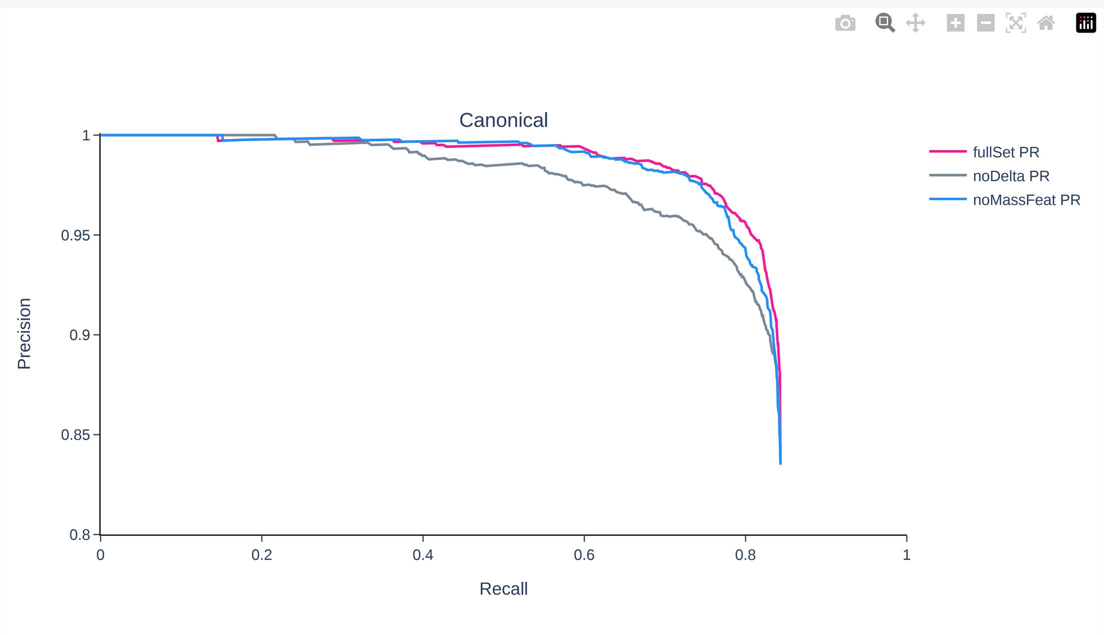

Receiver-Operator Curves

This plots the true positive rate (correct identifications divided by correct identifications and missed identifications) against the false positive rate (incorrect identifications divided by incorrect identifications and unidentifiable scan for which no peptide was identified) for each identification method as we vary the scoring the threshold from the maximum to the minimum score in the data.

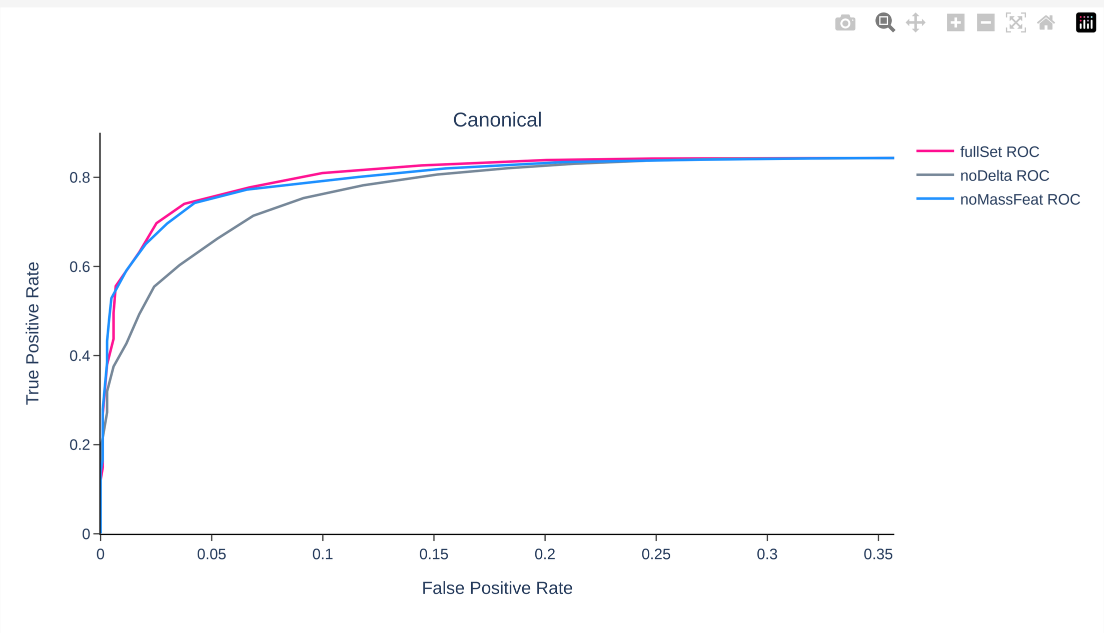

Score Distribution

This plots distributions of scores for correct, incorrect, and decoy PSMs for each stratum.

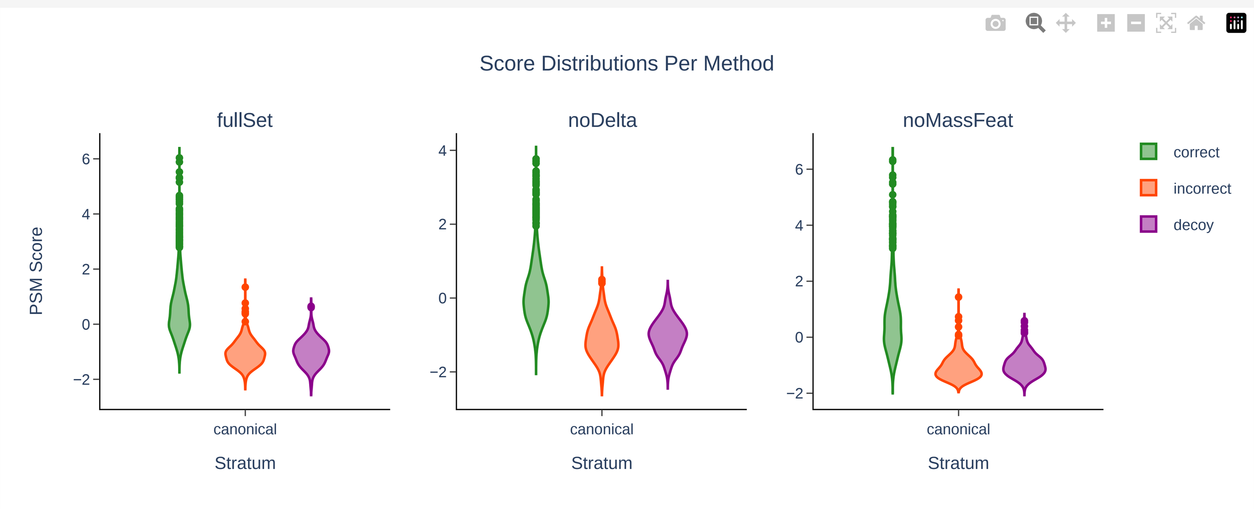

False Discovery Rate Estimation

This plots the estimated FDR against observed FDR for each method.

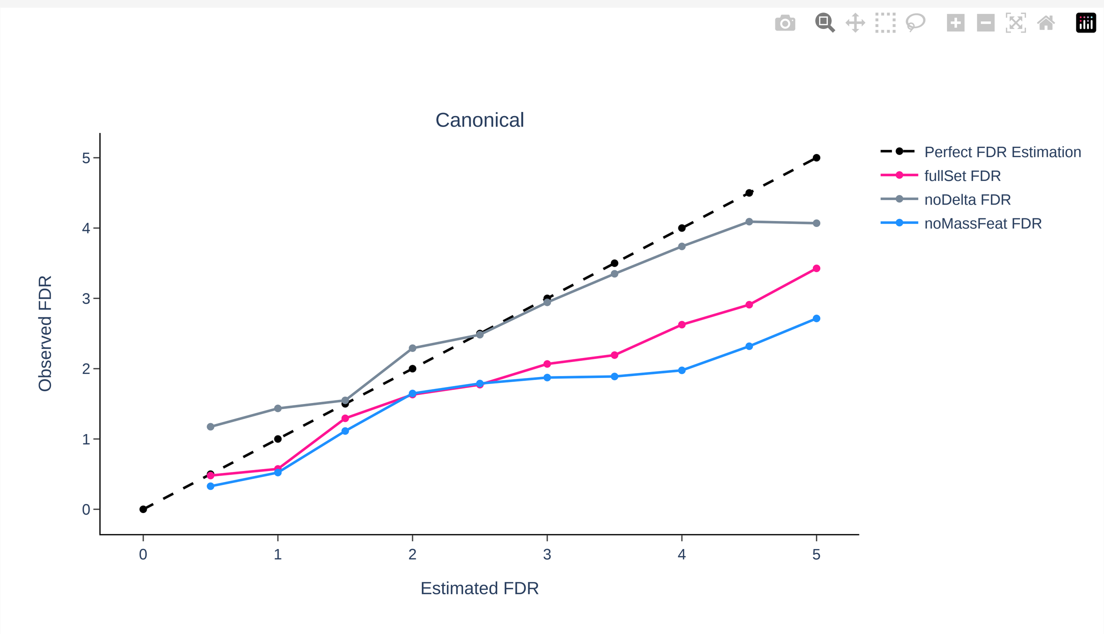

Confounding Variables

This plots search engine score distributions against the values of possible confounding variables for correct and incorrect PSMs for each identification method.

fullSet

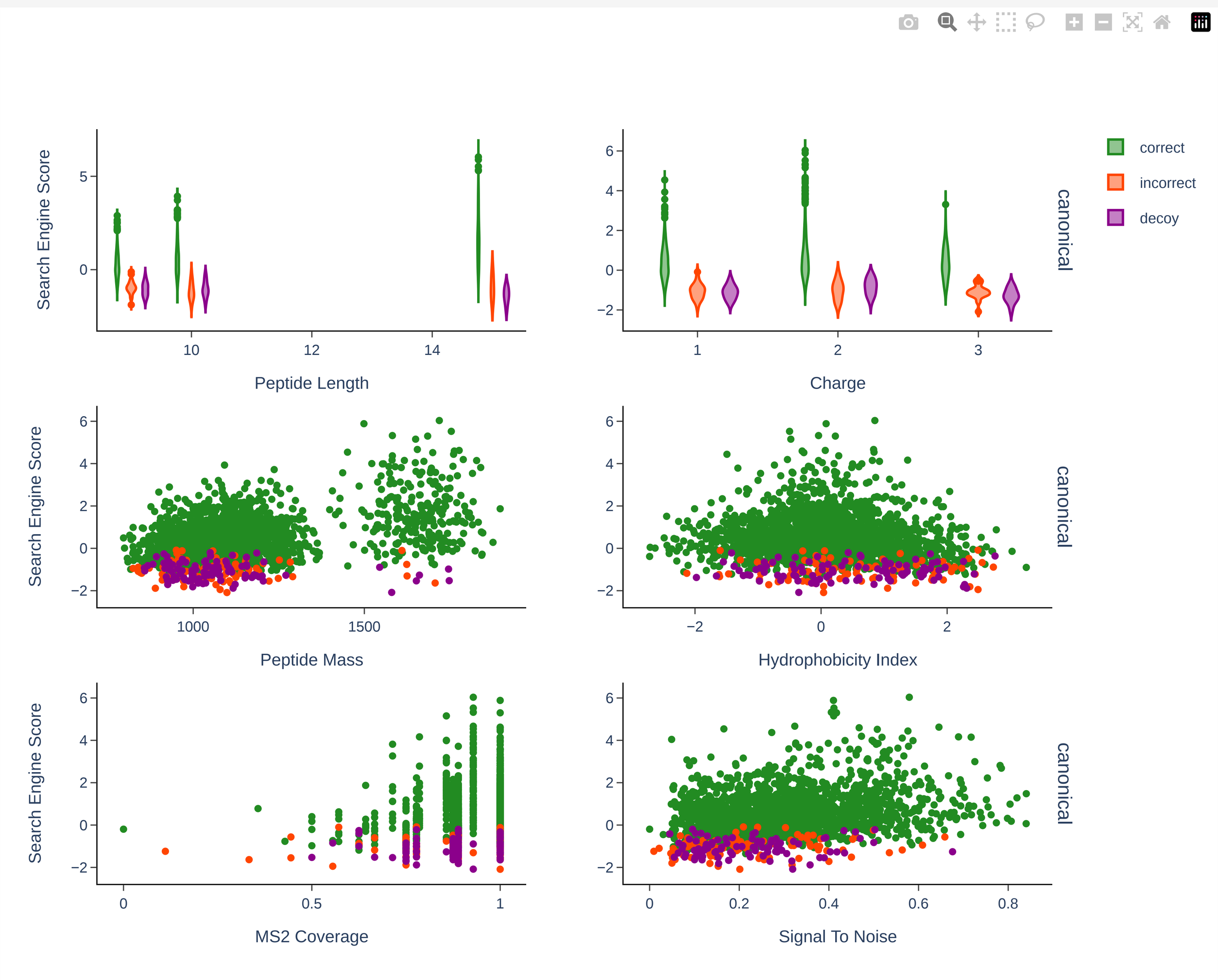

noDelta

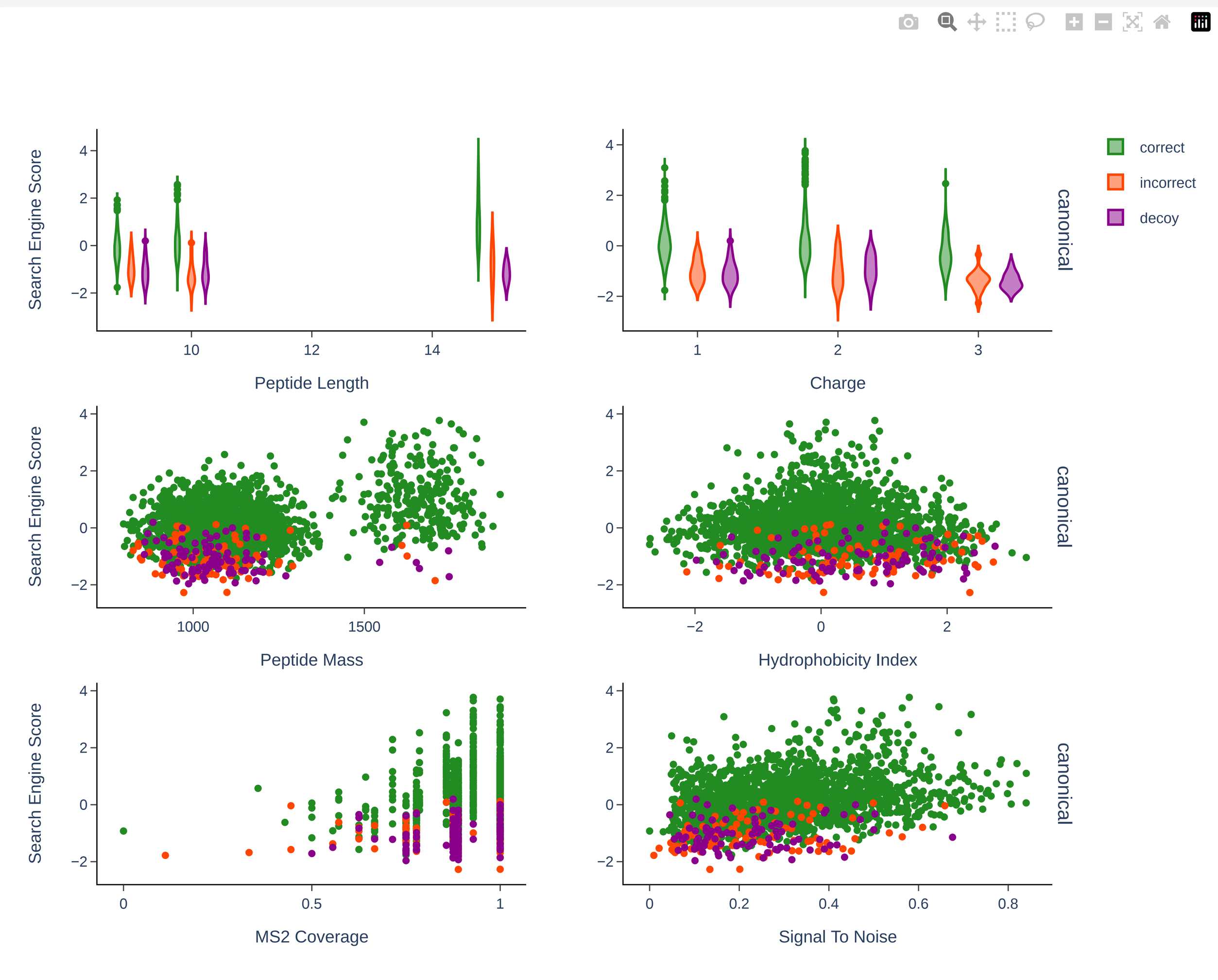

noMassFeat

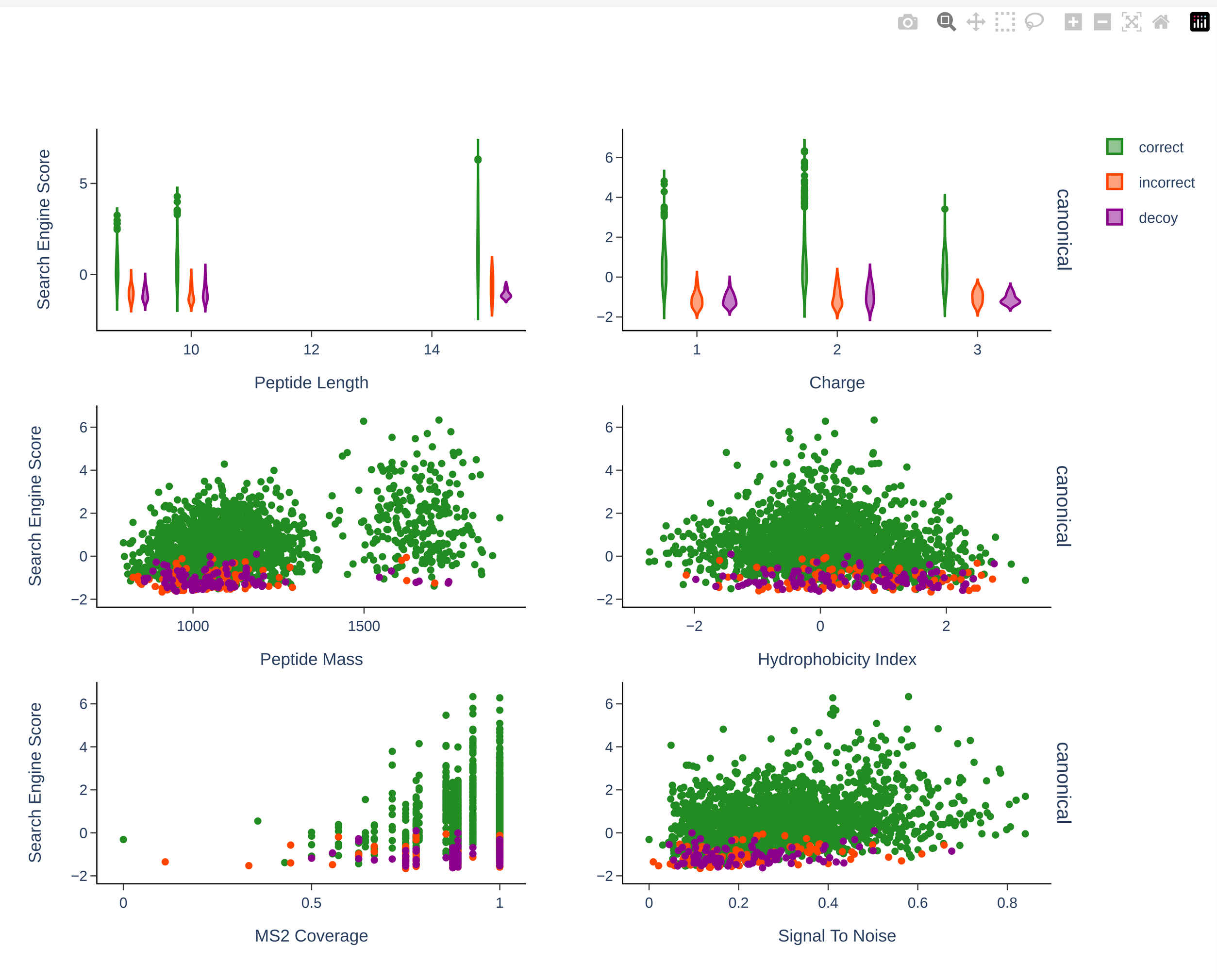

Supplement: Supplementary file 1 — Supporting Information [file PMIC-23-0-s001.pdf]
